# Translational Perspectives on Cell-Free Mitochondrial DNA as a Biomarker in Gynecological Cancers: Current Limitations and Future Research Directions

**DOI:** 10.3390/biom16060771

**Published:** 2026-05-25

**Authors:** Clara Musicco, Anna Signorile, Domenico De Rasmo, Vera Loizzi, Gennaro Cormio, Antonella Cormio

**Affiliations:** 1 Institute of Biomembranes, Bioenergetics and Molecular Biotechnologies (IBIOM), National Research Council of Italy (CNR), 70126 Bari, Italy; clara.musicco@cnr.it (C.M.); domenico.derasmo@cnr.it (D.D.R.); 2 Department of Translational Biomedicine and Neuroscience, University of Bari ‘Aldo Moro’, 70124 Bari, Italy; anna.signorile@uniba.it (A.S.); vera.loizzi@uniba.it (V.L.); 3 S.C. Ginecologia Oncologia Clinicizzata, IRCCS Istituto Tumori Giovanni Paolo II, 70124 Bari, Italy; gennaro.cormio@uniba.it; 4 Department of Interdisciplinary Medicine, University of Bari ‘Aldo Moro’, 70124 Bari, Italy; 5 Department of Precision and Regenerative Medicine and Ionian Area, University of Bari ‘Aldo Moro’, 70124 Bari, Italy

**Keywords:** liquid biopsy, cancer biomarkers, cell-free DNA, cell-free mitochondrial DNA, ovarian cancer, endometrial cancer

## Abstract

In recent years, liquid biopsy has emerged as a promising non-invasive strategy for the identification of tumor-derived biomarkers. Among circulating analytes, cell-free DNA (cfDNA), including both nuclear and mitochondrial fractions, has been extensively investigated in a variety of biological fluids for its potential applications in cancer diagnosis, disease monitoring, and prognostic stratification. Owing to its higher copy number per cell compared with nuclear DNA, mitochondrial DNA (mtDNA) is typically present at higher concentrations in body fluids and is therefore potentially detectable. Circulating cell-free mitochondrial DNA (cfmtDNA) is closely associated with pro-inflammatory signaling pathways and cellular damage responses, including apoptosis, necrosis, and neutrophil extracellular trap formation (NETosis). This review provides a comprehensive overview of the reported alterations of cfmtDNA in the most prevalent gynecological malignancies, namely ovarian and endometrial cancers, which are characterized by a chronic inflammatory microenvironment. We further critically assess the current evidence supporting cfmtDNA as a potential non-invasive biomarker in these malignancies, highlighting current limitations and future research directions.

## 1. Introduction

Tissue biopsy remains the standard approach for cancer diagnosis; however, it is inherently invasive. In recent years, the analysis of specific biomarkers in liquid biopsies, including serum, plasma, saliva, ascitic fluid, and urine, has emerged as a promising and non-invasive strategy for early cancer detection, accurate prognosis, and evaluation of therapeutic response [1,2].

**Figure 1:**
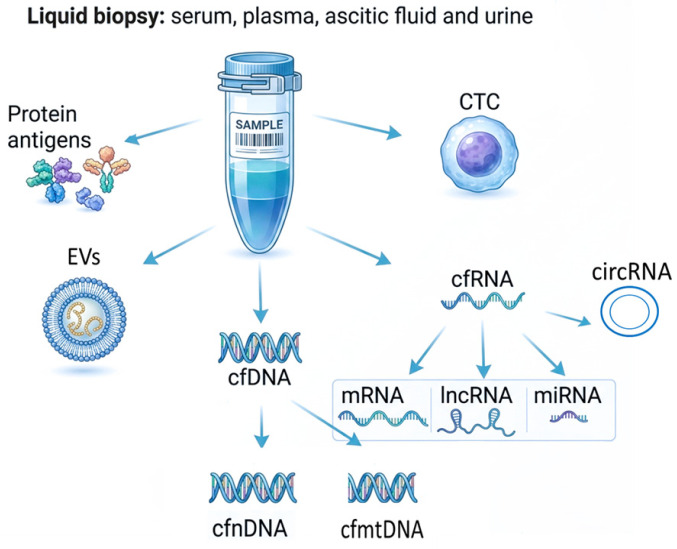
Analytes analyzed in liquid biopsy: protein antigens; CTC, cancer tumor cell; EVs, extracellular vesicles; cfRNA, cell-free RNA; mRNA, messenger RNA; lncRNA, long non-coding RNA; miRNA, microRNA; circRNA, circular RNA; cfDNA, cell-free DNA; cfnDNA, cell-free nuclear DNA; cfmtDNA, cell-free mitochondrial DNA.

Liquid biopsy analytes include cell-free DNA (cfDNA), protein antigens, circulating tumor cells (CTCs), cell-free RNA (cfRNA), and extracellular vesicles (EVs) [2]. Based on their cellular origin, cfDNA can be classified as cell-free nuclear DNA (cfnDNA) or cell-free mitochondrial DNA (cfmtDNA). Cell-free RNA comprises messenger RNA (mRNA), microRNA (miRNA), circular RNA (circRNA) and long non-coding RNA (lncRNA) [2,3,4,5]. Figure 1 summarizes the main classes of analytes investigated in liquid biopsy.

CfmtDNA is preferentially associated with pro-inflammatory stimulation [6], a key driver of tumor development, particularly in gynecological malignancies [7,8].

Ovarian and endometrial cancers account for a substantial proportion of cancer-related deaths among women worldwide. The lack of specific symptoms in the early stages of these diseases, together with the limited availability of reliable diagnostic tests, often results in delayed diagnosis. Moreover, these malignancies exhibit heterogeneous histological and metabolic phenotypes, which are associated with variable responses to therapy [9,10,11]. Notably, both ovarian and endometrial cancers are characterized by a pronounced inflammatory tumor microenvironment [8,12,13,14], supporting a potential role for cfmtDNA in cancer progression.

This review provides a comprehensive and critical evaluation of the current evidence regarding alterations in cfmtDNA in ovarian and endometrial cancers. We examine the available data supporting the potential role of cfmtDNA as a non-invasive biomarker for early detection, prognostic stratification, and disease monitoring in these malignancies, while also addressing methodological limitations and existing knowledge gaps.

## 2. Cell-Free DNA

CfDNA, including both mitochondrial and nuclear DNA, consists of short DNA fragments (approximately 120–200 bp) released into body fluids because of physiological cell turnover, including apoptosis and necrosis [15,16]. Accordingly, cfDNA originates from nuclease-mediated cleavage of cellular DNA derived from different cell types. In addition to passive release, DNA fragments can also be actively secreted by cells. The production and clearance of cfDNA are highly dynamic processes, resulting in a short half-life ranging from 5 to 150 min [16,17]. Plasma from healthy individuals contains measurable levels of cfDNA, the vast majority of which originates from leukocytes, particularly neutrophils, B cells, and T cells [18].

**Figure 2:**
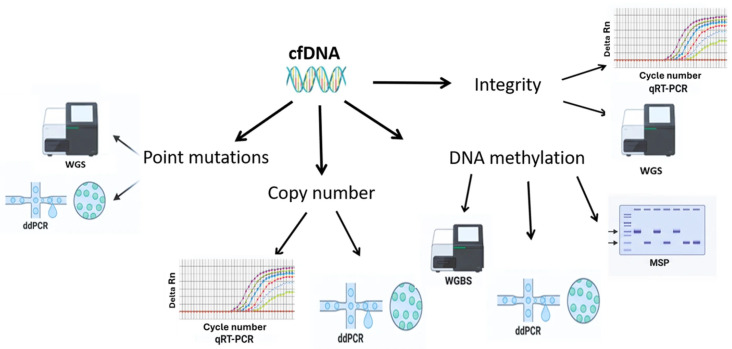
Schematic representation of analytical methods for cfDNA. Point mutations are detected by whole-genome sequencing (WGS) and droplet digital PCR (ddPCR); copy number variations are assessed using qRT-PCR and ddPCR; DNA methylation patterns are analyzed through whole-genome bisulfite sequencing (WGBS), ddPCR and methylation-specific PCR (MSP); and cfDNA integrity is evaluated by qRT-PCR and WGS.

CfDNA exhibits a wide range of alterations under both physiological and pathological conditions, including cancer. These alterations encompass mutations, variations in copy number and integrity, as well as aberrant DNA methylation patterns [19,20,21,22,23,24,25,26]. Given the low abundance of cfDNA in body fluids, analytical methods used to detect and characterize cfDNA alterations must be highly sensitive [3,27,28]. An overview of these methodologies is provided in Figure 2. Whole-genome sequencing (WGS) and droplet digital PCR (ddPCR) are employed for the detection of specific genetic mutations. Quantitative real-time PCR (qRT-PCR) and ddPCR are used to quantify cfDNA copy number, while qRT-PCR and WGS are used to assess cfDNA integrity. Finally, methylation-specific PCR (MSP), ddPCR, and whole-genome bisulfite sequencing (WGBS) are utilized to investigate cfDNA methylation patterns.

Interestingly, in several malignancies, cfDNA is detected at elevated concentrations in body fluids, and its levels often correlate with tumor grade, stage, and patient prognosis [18,27,29]. However, the majority of cfDNA in cancer patients does not originate from neoplastic cells or adjacent non-neoplastic epithelial cells, but rather from leukocytes, predominantly neutrophils, similar to what is observed in healthy individuals [18]. Several studies in oncology have focused specifically on circulating tumor DNA (ctDNA), a fraction of cfDNA released by tumor cells that harbors tumor-specific genetic alterations [4]. CtDNA fragments are typically ~146 base pairs in length and exhibit an even shorter half-life than total cfDNA [17]. Owing to its extremely low abundance in body fluids, ctDNA remains technically challenging to detect and accurately quantify.

### 2.1. Cell-Free Mitochondrial DNA

MtDNA is a circular, double-stranded molecule of approximately 16,569 base pairs. It encodes the 12S and 16S ribosomal RNAs, 22 transfer RNAs, and 13 protein subunits of the respiratory chain complexes, whereas the remaining mitochondrial proteins are encoded by nuclear DNA [30,31]. Each mitochondrion contains hundreds to thousands of mtDNA copies, in contrast to the two copies of nuclear DNA per cell. This feature, known as polyploidy, allows mtDNA molecules within a cell or tissue to exist either as a uniform population (homoplasmy), comprising exclusively wild-type or mutant genomes, or as a mixed population (heteroplasmy) [32,33].

MtDNA is more prone to mutations, including point mutations and deletions, than nuclear DNA. This increased mutational burden is related to continuous exposure to reactive oxygen species (ROS), which are mainly produced by the mitochondrial respiratory chain, the absence of protective histones, and a relatively inefficient DNA damage repair system [34]. Somatic mtDNA mutations have been reported across multiple cancer types [34,35].

Comparative analyses of cfmtDNA and cfnDNA have shown that cfmtDNA exhibits a distinct fragment size distribution, characterized by a smaller median fragment length (approximately 128 bp versus 166 bp). This difference may be due to the lack of histone association, which results in reduced protection of mtDNA from enzymatic degradation [36,37]. Furthermore, cfmtDNA fragments display a lower frequency of 5′ cytosine (C) and thymine (T) termini and a higher frequency of 5′ guanine (G) and adenine (A) termini. These observations suggest that the fragmentomic profile of cfmtDNA is closely linked to the unique structure, base composition, and protein-binding properties of mtDNA. In the context of liquid biopsy, cfmtDNA is generally easier to detect than cfnDNA due to its shorter fragment length and higher copy number [38].

Caicedo et al. [38] proposed a classification of cfmtDNA into four main categories: (i) naked mtDNA, present as whole molecules or fragments, either free or associated with proteins in nucleoid-like structures; (ii) exM-mtDNA, consisting of mtDNA enclosed within active or inactive extracellular mitochondria; (iii) M-mtDNA, referring to mtDNA contained within EVs, including exosomes; and (iv) MM-mtDNA, representing mtDNA enclosed within mitochondria or membranous structures and apoptotic bodies outside the cell. Naked mtDNA is predominantly associated with pro-inflammatory signaling and cellular damage responses, including apoptosis and necrosis, whereas exM-mtDNA and MM-mtDNA may contribute to intercellular communication and the maintenance of cellular homeostasis.

#### CfmtDNA and Inflammation

It is widely accepted that eukaryotic mitochondria originated from the permanent endosymbiosis of α-proteobacteria [39]. Mitochondrial components released from damaged or stressed cells, including proteins and mtDNA, are collectively referred to as mitochondrial damage-associated molecular patterns (mtDAMPs). Owing to their evolutionary similarity to bacterial products, mtDAMPs can trigger innate immune and pro-inflammatory responses in the absence of infection, a process known as sterile inflammation, primarily through activation of the Toll-like receptor 9 (TLR9) signaling pathway [40]. Not all mtDAMPs are passively released following cellular damage; some mitochondrial components are instead selectively packaged and released through autophagy-related mechanisms.

Elevated levels of cfmtDNA have been reported in various pathophysiological conditions, including psychological stress, inflammation, increased body mass index (BMI), cancer, and aging [41,42,43,44,45]. Increased plasma cfmtDNA levels have also been observed in patients with premature ovarian insufficiency (POI), a condition characterized by a chronic inflammatory state [46]. Moreover, even during healthy pregnancy, which is considered a physiological inflammatory condition due to placental apoptosis, elevated circulating cfmtDNA levels have been detected in maternal blood [40].

## 3. Ovarian Cancer

Ovarian cancer (OC) is the seventh-most common cancer worldwide and accounts for more deaths than any other malignancy of the female reproductive tract. Late diagnosis and the development of drug resistance are considered the primary contributors to the high mortality associated with OC [47].

OC is classified into two main categories: epithelial tumors, which account for approximately 95% of cases, and non-epithelial tumors [48,49]. Epithelial ovarian cancer includes high-grade serous carcinoma, clear cell carcinoma, endometrioid carcinoma, mucinous carcinoma, and low-grade serous carcinoma. The etiology of OC is multifactorial and is frequently associated with genetic and epigenetic alterations, including oncogene activation, loss of tumor suppressor gene function, and dysregulation of key cell signaling pathways [50].

The tumor microenvironment of OC is characterized by a complex interplay of inflammatory and immunosuppressive processes, including the accumulation of ascites [51]. Within this microenvironment, cancer cells release signaling molecules such as chemokines and cytokines that recruit neutrophils to the tumor site, where they promote the formation of neutrophil extracellular traps (NETs) [52,53]. NETs are web-like extracellular structures primarily composed of neutrophil-derived DNA that function to trap and eliminate pathogens.

The most widely used biomarker for the diagnosis and monitoring of treatment response and drug resistance in OC remains serum cancer antigen 125 (CA125) [54,55]. CA125 exhibits limited sensitivity in early-stage disease (approximately 50%), although sensitivity increases to around 80% in advanced stages [54]. However, its diagnostic accuracy is limited by low specificity, as it can be elevated in several benign gynecological conditions, including endometriosis [56,57]. In addition to CA125, Human Epididymis Protein 4 (HE4) has emerged as a relevant biomarker for ovarian cancer detection. HE4 has been shown to provide higher specificity for ovarian malignancies and is less frequently elevated in benign conditions. The combined use of CA125 and HE4 improves diagnostic performance compared to either marker alone. This approach is implemented in the Risk of Ovarian Malignancy Algorithm (ROMA), which incorporates serum biomarker levels and menopausal status to stratify patients into different risk categories [57,58,59]. Consequently, the identification and validation of novel biomarkers, particularly in combination with existing markers, may improve the accuracy of OC diagnosis and prognostic stratification.

### 3.1. CfmtDNA as a Diagnostic Marker in Ovarian Cancer

CfmtDNA levels were quantified by qRT-PCR in serum and plasma samples obtained from 24 patients with benign ovarian tumors, 21 patients with epithelial ovarian cancer (EOC), 23 patients with endometriosis, and 36 age-matched healthy controls [60]. Plasma cfmtDNA levels were significantly elevated in patients with EOC compared with healthy women and those with benign epithelial ovarian tumors or endometriosis. Receiver operating characteristic (ROC) curve analysis demonstrated that plasma cfmtDNA effectively discriminates the ovarian cancer group from the healthy control group with a sensitivity of 63% and a specificity of 67% (AUC: 0.71, 95% CI: 0.569–0.852). Furthermore, no significant associations were found between cfmtDNA levels and tumor stage, tumor volume, or serum CA125 concentrations. No association was observed between cfmtDNA and cfnDNA levels, suggesting distinct mechanisms of release. Total serum cfDNA (including both mitochondrial and nuclear fractions) was significantly higher than plasma cfDNA across all study groups; however, no significant differences between groups were detected. The elevated cfDNA levels observed in serum are likely attributable to pre-analytical artifacts related to the coagulation process, which may promote cellular DNA release during clot formation. These findings indicate that plasma-derived cfmtDNA may serve as an independent diagnostic biomarker in EOC.

The levels of mtDNA in whole blood and plasma, including both cell-free and exosomal mtDNA fractions, were quantified by qRT-PCR in 24 patients with serous EOC and 24 healthy controls [61]. Exosomes are extracellular vesicles with a diameter of approximately 100 nm that contain nucleic acids, proteins, lipids, amino acids, and metabolites. Whole-blood mtDNA copy number was significantly reduced in cancer patients, both at early and advanced stages, compared with controls. In contrast, exosomal mtDNA copy number was significantly increased in patients with advanced-stage disease (FIGO stages III–IV), whereas no significant increase was observed in stage I disease. Plasma cfmtDNA levels did not differ significantly between patients and healthy individuals. The authors proposed that reduced whole-blood mtDNA copy number may serve as a biomarker of early-stage serous EOC, while increased exosomal mtDNA levels in advanced disease may contribute to metastatic dissemination and reflect tumor progression.

CfmtDNA integrity was evaluated in serum samples from 165 EOC patients and 60 healthy women using qRT-PCR [62]. MtDNA integrity was defined as the ratio of long (230 bp) to short (79 bp) fragments within the mitochondrial *16S rRNA* gene. Compared with healthy controls, EOC patients exhibited significantly reduced mtDNA integrity, potentially reflecting increased apoptotic activity. Absolute levels of both cfmtDNA79 and cfmtDNA230 fragments were significantly elevated in EOC patients. Among them, cfmtDNA79 showed good diagnostic performance, distinguishing patients from controls with 90.3% sensitivity and 81.7% specificity (AUC = 0.900). When stratified by FIGO stage, cfmtDNA79 and cfmtDNA230 levels increased progressively from healthy controls to early-stage (FIGO I–II) and advanced-stage (FIGO III–IV) disease, indicating a relationship with tumor progression. Higher levels were also associated with lymph node metastasis and increased CA125 levels. Overall, these results suggest that reduced cfmtDNA integrity and increased circulating mtDNA79 and mtDNA230 levels are associated with more advanced EOC and may reflect disease progression.

Collectively, the available data indicate that the diagnostic potential of circulating cfmtDNA in EOC remains insufficiently defined owing to the limited body of evidence currently available.

### 3.2. CfmtDNA as a Prognostic Marker in Ovarian Cancer

Given that the volume of ascites at initial diagnosis is associated with poorer progression-free survival (PFS) and overall survival (OS) in EOC, cfmtDNA levels were quantified by qRT-PCR in 68 ascitic fluid samples [63]. The highest quartile of ascites mtDNA was associated with reduced PFS and a higher likelihood of disease progression within 12 months following primary surgery (*n* = 68, log-rank *p* = 0.0178). The authors hypothesized that ascitic cfmtDNA may promote NETosis, thereby facilitating metastatic dissemination and suppressing antitumor immune responses.

The prognostic value of cfmtDNA integrity was evaluated in serum samples from 165 EOC patients [62]. The median follow-up time was 16 months, while the median overall and recurrence-free survival times were 16 and 14 months, respectively. While cfmtDNA integrity per se was not significantly associated with prognosis, Kaplan–Meier survival analysis and log-rank testing demonstrated that higher levels of mtDNA79 (*p* = 0.0001; HR 3.2, 95% CI: 1.6–6.3) and mtDNA230 (borderline *p* = 0.048, HR 0.9, 95% CI: 0.9–1.0) were associated with poor patients’ OS. Notably, only mtDNA79 emerged as an independent predictor of OS. These findings may reflect the increased release of mtDNA79 fragments into the circulation as a consequence of extensive tumor and stromal cell death in advanced disease.

More recently, a two-center prospective study including 188 patients with newly diagnosed EOC evaluated cfmtDNA levels in both serum and ascites using qRT-PCR and associated these measurements with clinical outcomes, including PFS and OS [64]. Although cfmtDNA levels were higher in ascitic fluid than in serum, no significant associations were observed between cfmtDNA concentrations and clinical outcomes in either biological compartment.

The prognostic role of cfmtDNA levels in OC has yet to be fully defined. Large, well-designed prospective studies are needed to clarify its potential.

### 3.3. CfmtDNA and Therapeutic Response in Ovarian Cancer

CfmtDNA levels were quantified by qRT-PCR in plasma samples from 100 untreated patients with advanced EOC and from 20 patients following debulking surgery and chemotherapy [65]. Post-treatment cfmtDNA levels showed a modest, non-significant decrease compared with baseline values. Median pre-treatment levels of total cfDNA and cfnDNA were higher in patients with more advanced disease, and post-therapy levels of both markers decreased significantly relative to baseline. Based on these findings, the authors suggested that total cfDNA and cfnDNA, but not cfmtDNA, may serve as indicators of therapeutic response. However, the limited number of post-treatment samples, together with the absence of analyses correlating cfmtDNA levels with PFS and OS, restricts the interpretation of its prognostic significance in this context.

In a subsequent study, plasma cfmtDNA levels were measured in 67 ovarian cancer patients before and after debulking surgery followed by six cycles of chemotherapy [66]. CfmtDNA levels decreased significantly after treatment, with a reduction of approximately 52%. Patients with stage I disease exhibited significantly lower baseline cfmtDNA levels compared with those with stage II–IV disease. However, pre-treatment cfmtDNA levels were not predictive of either PFS or OS. Notably, patients with higher post-treatment cfmtDNA levels experienced significantly shorter PFS than those with lower post-treatment levels. These findings suggest that a treatment-related reduction in cfmtDNA may reflect therapeutic efficacy and that post-treatment cfmtDNA levels may have prognostic value in ovarian cancer. Well-powered, rigorously designed prospective studies are required to define the role of cfmtDNA as a predictive biomarker of therapeutic response.

A summary of the above-reported studies on the role of cfmtDNA as diagnostic, prognostic and predictive marker in EOC patients is provided in Table 1.

The inconsistencies observed across studies may be attributable to: (1) small sample sizes; (2) the heterogeneity of the analyzed cohorts, which often include patients with different EOC subtypes (e.g., serous, endometrioid, other non-specified subtypes); (3) the influence of non-tumor-related factors, such as systemic inflammation or comorbid conditions, (4) the use of different cfmtDNA extraction methods; and (5) variability in qRT-PCR analyses, including differences in the targeted nuclear and mitochondrial genes, the chemistries employed (e.g., SYBR Green versus TaqMan probe-based assays), and the approaches used to calculate mtDNA levels. Additionally, methodological details, particularly those concerning plasma and serum preparation, are not consistently reported across all studies.

## 4. Endometrial Cancer

Endometrial cancer (EC) is one of the most common gynecological malignancies, and its incidence has increased markedly over the past few decades [67]. The development of EC is strongly associated with metabolic and hormonal risk factors, including metabolic syndrome, obesity, diabetes, low levels of physical activity, nulliparity, early menarche, late menopause, increasing age, and the use of unopposed hormone replacement therapy [68].

The etiology of EC is multifactorial and frequently involves both genetic and epigenetic alterations. Epigenetic dysregulation in EC is characterized by aberrant DNA methylation at CpG islands, largely driven by the activation of DNA methyltransferases [69,70,71]. In addition, recurrent genetic alterations have been described in EC, including microsatellite instability, mutations in the exonuclease domain of POLE, alterations in tumor suppressor genes such as *PTEN* and *TP53*, and mutations in oncogenes including *KRAS*, *CTNNB1* (β-catenin), and *ARID1A* [70,72].

Chronic low-grade inflammation has also been implicated in EC pathogenesis and may partly explain the strong association between obesity and EC risk. In line with this hypothesis, elevated circulating levels of pro-inflammatory biomarkers, including C-reactive protein (CRP), interleukin-6 (IL-6), and interleukin-1 receptor antagonist (IL-1Ra), have been associated with an increased risk of endometrial cancer [73]. Moreover, the tumor microenvironment in EC releases soluble mediators, including chemokines and cytokines, that recruit neutrophils to the tumor site, thereby promoting the formation of NETs.

Currently, histopathological examination of tissue or biopsy samples, procedures that are invasive and often painful, remains the gold standard for EC diagnosis and prognostic assessment. Late diagnosis and drug resistance remain major contributors to EC-related mortality [74]. Consequently, there is a pressing need to develop more sensitive and non-invasive biomarkers to enable early diagnosis and monitor therapeutic response in patients with EC [75].

### Cell-Free mtDNA as a Diagnostic Marker in Endometrial Cancer

Serum levels of cfmtDNA were quantified by qRT-PCR in 22 healthy subjects and 59 patients with EC prior to surgery and the initiation of any treatment, and were correlated with clinical characteristics, including tumor grade and stage, body mass index (BMI), age, hypertension, and inflammatory markers [76]. CfmtDNA levels did not significantly change between the two samples groups. ROC analysis confirmed the poor predictive capability of the relative cfmtDNA content to discriminate between healthy subjects and EC patients (AUC equal to 0.51). No significant association was found between cfmtDNA and tumor stage, grade, BMI and age. Regarding total cfDNA, significantly higher concentrations were detected in the serum of EC patients compared with healthy subjects, with the most pronounced increases observed in high-grade EC. The authors hypothesized that the inflammatory and hypoxic tumor microenvironment may promote neutrophil recruitment and the formation of tumor-associated neutrophils, which in turn stimulate DNA release through NETosis. Given that neutrophils contain relatively few mitochondria compared with other leukocytes, this mechanism may explain the increase in total cfDNA without a corresponding rise in mtDNA levels.

In a subsequent study including a larger cohort of EC patients, cfmtDNA levels were quantified by qRT-PCR in deactivated serum samples from 63 EC patients and 21 healthy controls [77]. This analysis, revealed a significant decrease in cfmtDNA levels in EC patients compared with healthy subjects. ROC curve analysis identified threshold values for cfmtDNA that effectively discriminated EC patients from controls with a sensitivity of 65% and a specificity of 95% (AUC: 0.854, 95% CI: 0.77–0.94) *p* < 0.001. Conversely, cfDNA levels significantly increased in EC patients and were able to discriminate EC patients from controls. Consistent with previous observations [76], the authors reported evidence of NETosis in EC tissues. Citrullinated histone H3 (citH3), a recognized biomarker of NETosis indicating the release of chromatin, was significantly associated with total cfDNA levels and inversely associated with cfmtDNA levels. These findings indicate that reduced serum cfmtDNA and increased cfDNA levels may represent novel, non-invasive biomarkers of EC, particularly reflecting inflammatory processes associated with NETosis. Compared with the previous report, serum deactivation may explain the different results. A summary of the above-reported studies on the role of cfmtDNA as a diagnostic marker in EC patients is reported in Table 2.

## 5. Discussion and Challenges

In recent years, studies investigating cfmtDNA alterations in body fluids from OC and EC patients, including serum, plasma, and ascites, have yielded promising, although still limited, evidence supporting its potential as a biomarker for these malignancies. Nevertheless, some findings remain controversial.

Interestingly, the increased abundance of cfmtDNA79 and cfmtDNA230 16S rRNA fragments in OC has been associated with advanced disease stage and reduced OS, underscoring their potential diagnostic and prognostic value [62]. Furthermore, OC patients with persistently elevated post-treatment cfmtDNA levels appear to exhibit shorter PFS compared with those showing lower levels [66]. Collectively, these findings suggest that, in OC, the accumulation of cfmtDNA, particularly the short cfmtDNA79 fragment, together with increased cfnDNA levels, may reflect enhanced NETosis activity.

The interplay between NETs and OC progression is increasingly recognized as a critical axis in tumor biology [78]. Three principal forms of NETosis have been described [8,53,79]: (i) suicidal NETosis, a form of neutrophil cell death distinct from apoptosis and necrosis, characterized by nuclear membrane rupture and chromatin release; (ii) vital NETosis, involving vesicle-mediated chromatin extrusion without immediate cell death; and (iii) mitochondrial NETosis, driven by mitochondrial ROS production and the release of oxidized mtDNA from viable neutrophils.

**Figure 3:**
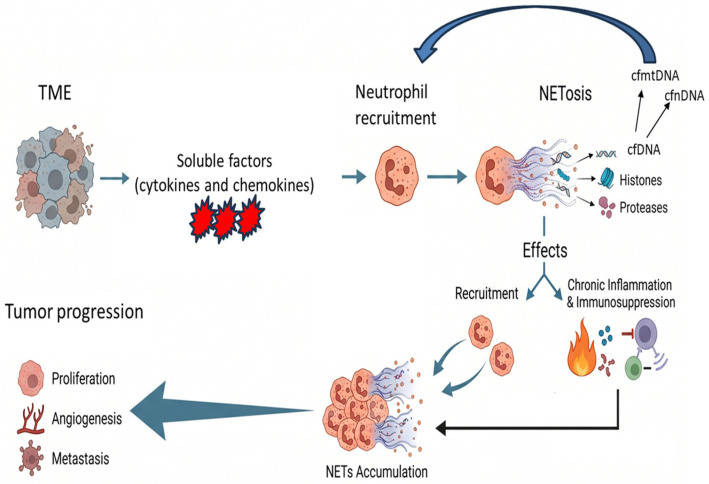
The ovarian cancer microenvironment induces NETosis to promote tumor growth, metastasis, and immune evasion. The tumor microenvironment (TME) releases soluble factors that recruit neutrophils and induce NETosis, leading to the release of cfDNA (cfmtDNA and cfnDNA), histones, and proteases. CfmtDNA may amplify inflammation and immunosuppression and promote tumor progression (proliferation, angiogenesis, and metastasis).

It can be hypothesized that the ovarian tumor microenvironment (TME) secretes a variety of soluble factors, including chemokines and inflammatory cytokines, which promote neutrophil recruitment and induce both suicidal and mitochondrial NETosis. This process leads to the release of cfDNA, particularly cfmtDNA, as well as histones and proteases. CfmtDNA may amplify a feed-forward cycle of inflammation and immunosuppression, thereby facilitating NET-mediated tumor progression, including enhanced proliferation, angiogenesis, and metastasis. An overview of this hypothesis is shown in Figure 3.

Increased oxidative stress and reduced antioxidant capacity have been reported in the peripheral blood of patients with OC and are associated with elevated cytokine levels, neutrophil infiltration and enhanced NETosis [52,80]. These conditions may contribute to the increased release of cfmtDNA. Effective treatment may attenuate inflammation, NETosis, and cfmtDNA release, thereby contributing to a more favorable clinical outcome. Although NETs represent promising therapeutic targets, their dual role as both protective immune effectors and pro-tumorigenic mediators underscores the complexity of therapeutically modulating this pathway.

In EC, it can be hypothesized that the observed decrease, or lack of change, in serum circulating cfmtDNA levels, alongside a concomitant increase in cfnDNA [76,77], may reflect inflammation-driven suicidal or vital NETosis during disease progression. In this context, activated neutrophils may preferentially release nDNA, with only a limited contribution from mtDNA extrusion. Furthermore, neutrophils contain relatively few mitochondria compared with other leukocyte subsets, which could further explain the reduced cfmtDNA levels observed. Future studies should aim to elucidate the mechanisms underlying NET formation in EC and OC, including the identification of upstream triggers and downstream effects.

However, results across studies are sometimes contradictory. Such discrepancies may be attributable to methodological differences in sample collection and processing, which can substantially influence quantitative measurements [81,82]. In particular, variations in cfmtDNA levels may arise from the use of serum rather than plasma; therefore, plasma is generally preferred to minimize pre-analytical artifacts associated with platelet activation during coagulation. Additionally, cfmtDNA quantification may vary depending on the anticoagulant used for plasma collection, such as heparin, EDTA, or citrate.

Reliable analysis of cfmtDNA critically depends on its enrichment through optimized pre-analytical workflows designed to minimize cellular contamination and maximize recovery of circulating mitochondrial fragments. Plasma should undergo double centrifugation to remove residual cells and platelets, which represent major sources of contaminating mtDNA. In parallel, extracellular vesicle isolation, via differential ultracentrifugation and/or size-exclusion chromatography, is essential to enrich for vesicle-associated cfmtDNA, a biologically relevant circulating fraction [82,83]. Strict standardization of sample handling, storage, and freeze–thaw conditions is required to reduce variability in cfmtDNA measurements [82,84,85].

On the analytical level, highly sensitive platforms such as digital PCR and next-generation sequencing are expected to further improve the quantification and molecular characterization of cfmtDNA [85]. Digital PCR has emerged as the reference method for cfmtDNA quantification due to its absolute measurement capability and resistance to PCR inhibition, with detection limits approaching 1 copy/μL. These two approaches will enable not only the quantification of circulating mtDNA levels but also the assessment of fragmentomics, integrity indices, and potential mutation profiles, thereby expanding the biological and clinical information content of cfmtDNA. The reliability of cfmtDNA measurement is further complicated by mtDNA hypervariability and the presence of nuclear mitochondrial DNA sequences (NUMTs), which result from the integration of mitochondrial genome fragments into nuclear DNA [86]. Consequently, amplification strategies should target mtDNA regions characterized by low mutation rates and minimal homology with nuclear DNA.

CfmtDNA has been increasingly investigated across multiple tumor types, including testicular, breast, hepatocellular and lung cancers, supporting its broader relevance in the liquid biopsy landscape [87,88,89,90]. CfmtDNA levels and fragmentomic features may correlate with tumor burden, disease progression, and multi-cancer detection [37,89,90]. However, because results remain partly inconsistent, the need for standardized pre-analytical and analytical conditions likely extends beyond gynecological cancers and reflects broader limitations in the clinical application of cfmtDNA.

## 6. Conclusions

In ovarian and endometrial cancer, the combined assessment of cfmtDNA levels, NET-associated biomarkers (such as cfDNA–neutrophil complexes, myeloperoxidase–DNA complexes, or citrullinated histone H3), and established clinical markers (CA125, HE4, and ROMA) may provide a more comprehensive representation of tumor biology by capturing both tumor-derived and host immune-related components. CfmtDNA levels may also reflect tumor burden and disease aggressiveness and could have potential utility in disease monitoring, including the evaluation of treatment response and detection of recurrence.

In the future, artificial intelligence-based approaches integrating cfmtDNA, circulating tumor DNA, protein biomarkers, and imaging data may enable a more dynamic assessment of tumor behavior and support its integration into precision oncology workflows. However, its clinical application is currently limited by the lack of standardized pre-analytical and analytical procedures and the absence of validated cutoff values, which contribute to variability across studies. Further large-scale, prospective longitudinal studies are required to confirm its diagnostic and prognostic performance and support its translation into clinical practice.

## Figures and Tables

**Table 1:** CfmtDNA as a diagnostic, prognostic and predictive marker in OC patients.

Diagnostic Value					
Biomarker	Sample	Clinical Significance	N° Cases/Controls	Methods (Chemistry, Genes)	Ref.
CfmtDNA level	Plasma	Elevated levels differentiate EOC from healthy and benign groups	21 EOC, 24 BEOC, 23 endometriosis, 36 controls	qRT-PCR (TaqMan MGB probes, GAPDH MTATP8)	[60]
	
	Serum	No diagnostic value			
CfmtDNA level	Plasma	No diagnostic value	24 serous EOC, 24 controls	qRT-PCR (SYBR Green, SLCO2B1 SERPINA1 MTND1 MTND5)	[61]
	
	Whole blood	Decreased in EOC vs. control patients			
	
	Exosome	Increased in advanced disease vs. control patients			
CfmtDNA 79 and 230 pb level	Serum	Increased level in EOC and in cancer progression	165 EOC, 60 controls	qRT-PCR (SYBR Green, GAPDH MT16S)	[62]
	
CfmtDNA integrity		Reduced in EOC vs. control patients			
**Prognostic Value**					
CfmtDNA level	Ascites	Higher levels are associated with shorter PFS	68 EOC	qRT-PCR (SYBR Green, MT-CYB)	[63]
CfmtDNA 79 and 230 pb level	Serum	Higher levels are associated with reduced OS	165 EOC	qRT-PCR (SYBR Green, GAPDH MT16S)	[62]
	
CfmtDNA integrity		No association with prognosis			
CfmtDNA level	Ascites	No association with prognosis	188 EOC	qRT-PCR (SYBR Green, ACTB MT-CYB)	[64]
	
	Serum	No association with prognosis			
**Therapeutic Response**					
CfmtDNA level	Plasma	No significant change in post-treatment levels	20 EOC	qRT-PCR (SYBR Green, GAPDH MTATP 8)	[65]
CfmtDNA level	Plasma	Post-treatment reduction increased PFS	67 OC	qRT-PCR (SYBR Green, HGB MT-CYB)	[66]

EOC, epithelial ovarian cancer; BEOC, benign epithelial ovarian cancer; OC, ovarian cancer; OS, overall survival; PFS, progression-free survival; qRT-PCR, quantitative real-time PCR; vs., versus.

**Table 2:** CfmtDNA as a diagnostic marker in EC patients.

Biomarker	Sample	Clinical Significance	N° Cases/Controls	Methods (Chemistry, Genes)	Ref.
CfmtDNA level	Serum	No diagnostic value	59 EC, 22 controls	qRT-PCR (SYBR Green, HGB 36B4 MTND1 MT16S)	[76]
CfmtDNA level	Deactivated serum	Reduced in EC vs. control patients	63 EC, 21 controls	qRT-PCR (SYBR Green, 36B4 MTND1 MT16S)	[77]

EC, endometrial cancer; qRT-PCR, quantitative real-time PCR; vs., versus.

## Data Availability

No new data were created or analyzed in this study. Data sharing is not applicable to this article.
